# Aortic Pulse Wave Velocity as a Measure of Cardiovascular Risk in Chronic Obstructive Pulmonary Disease: Two-Year Follow-Up Data from the ARCADE Study

**DOI:** 10.3390/medicina55040089

**Published:** 2019-04-02

**Authors:** Nichola S. Gale, Ali M. Albarrati, Margaret M. Munnery, Barry J. Mcdonnell, Victoria S. Benson, Ruth M. Tal-Singer, John R. Cockcroft, Dennis J. Shale

**Affiliations:** 1 School of Healthcare Sciences, Heath Park Campus, Cardiff University, Cardiff CF14 4XN, UK; albarrati@ksu.edu.sa (A.M.A.); shaledj@cardiff.ac.uk (D.J.S.); 2 Rehabilitation Sciences Department, College of Applied Medical Sciences, King Saud University, Riyadh 11433, Saudi Arabia; 3 Department of Bio Medical Sciences, Cardiff School of Health Sciences, Cardiff Metropolitan University; Llandaff Campus, Western Ave, Cardiff CF5 2YB, UK; MMunnery@cardiffmet.ac.uk (M.M.M.); bmcdonnell@cardiffmet.ac.uk (B.J.M.); jcockcroft@cardiffmet.ac.uk (J.R.C.); 4 GSK Research and Development, GSK Stockley Park West, Uxbridge, Middlesex UB11 1BT, UK; victoria.x.tribble@gsk.com; 5 GSK Research and Development, Collegeville, PA 19426, USA; ruth.m.tal-singer@gsk.com

**Keywords:** COPD, comorbidities, cardiovascular risk, arterial stiffness, progression

## Abstract

*Background and objectives*: Cardiovascular (CV) disease is a major cause of morbidity and mortality in chronic obstructive pulmonary disease (COPD). Patients with COPD have increased arterial stiffness, which may predict future CV risk. However, the development of arterial stiffness in COPD has not yet been studied prospectively. The Assessment of Risk in Chronic Airways Disease Evaluation (ARCADE) is a longitudinal study of CV risk and other comorbidities in COPD. The aims of this analysis were to explore factors associated with aortic pulse wave velocity (aPWV) at baseline and to describe the progression of aPWV in patients with COPD and comparators over two years. *Materials and methods*: At baseline, 520 patients with COPD (confirmed by spirometry) and 150 comparators free from respiratory disease were assessed for body composition, blood pressure, aPWV, noninvasive measures of cardiac output, inflammatory biomarkers, and exercise capacity. This was repeated after two years, and mortality cases and causes were also recorded. *Results*: At baseline, aPWV was greater in COPD patients 9.8 (95% confidence interval (CI) 9.7–10) versus comparators 8.7 (8.5–9.1) m/s (*p* < 0.01) after adjustments for age, mean arterial pressure (MAP), and heart rate. Mean blood pressure was 98 ± 11 in COPD patients and 95 ± 10 mmHg in comparators at baseline (*p* = 0.004). After two years, 301 patients and 105 comparators were fully reassessed. The mean (95% CI) aPWV increased similarly in patients 0.44 (0.25–0.63) and comparators 0.46 (0.23–0.69) m/s, without a change in blood pressure. At the two-year follow-up, there were 29 (6%) deaths in COPD patients, with the majority due to respiratory causes, with an overall dropout of 43% of patients with COPD and 30% of comparators. *Conclusions*: This was the first large longitudinal study of CV risk in COPD patients, and we confirmed greater aPWV in COPD patients than comparators after adjustments for confounding factors. After two years, patients and comparators had a similar increase of almost 0.5 m/s aPWV.

## 1. Introduction

Chronic obstructive pulmonary disease (COPD) is now the third leading cause of mortality worldwide [1]. Although many associate COPD mainly with a progressive decline in lung function, this disease should be actually regarded as a multisystem disorder with major extrapulmonary complications and comorbidities. These include increased cardiovascular (CV) risk, loss of skeletal muscle mass and function, decreased exercise capacity, the development of osteoporosis, insulin resistance, and diabetes mellitus [2,3,4,5,6].

Cardiovascular disease is a major cause of mortality, accounting for 30–50% of deaths in COPD, independent of cigarette smoking status [7]. Aortic stiffness is an independent predictor of CV morbidity and mortality in general and diseased populations and has been related to a decline in the forced expiratory volume in one second (FEV_1_) [8,9]. It has been suggested that increased arterial stiffness causes premature vascular aging and contributes to excess CV disease in COPD [10]. Increased aortic stiffness occurs with mid-to-late central systolic pressure and leads to increasing left ventricular afterload and myocardial oxygen demand, while reducing diastolic pressure augmentation of myocardial perfusion and increasing the risk of ischemia and ventricular dysfunction. A cross-sectional population study has confirmed greater aortic pulse wave velocity (aPWV) in COPD using a Vicorder device, which was associated with reported CV disease but did not report or adjust for blood pressure [11]. These changes can present regardless of the severity of airflow limitation from COPD [12]. Aortic pulse wave velocity is a reliable measure of aortic stiffness and is increased in COPD [13]. Nevertheless, to date, no measure of arterial stiffness has been validated as a prognostic CV risk predictor in COPD.

The mechanism of CV risk in COPD has not been fully established, although there have been a number of suggestions for the development of increased arterial stiffness. Chronic systemic inflammation, increased insulin resistance, the risk of developing diabetes mellitus [3,6], as well as low fat-free mass and bone mineral density have been related to aortic stiffness in COPD in cross-sectional studies [10].

The progression of aPWV in COPD has not yet been studied prospectively: Such studies may help to elucidate the mechanism of CV risk. The Assessment of Risk in Chronic Airways Disease Evaluation (ARCADE) is a longitudinal study of CV risk and other comorbidities in COPD [13]. The primary aims are to delineate the progression and inter-relationships of CV disease and associated comorbidities. The aims of this analysis were to explore factors associated with aPWV at baseline and to describe the progression of aortic pulse wave velocity in patients with COPD and comparators over two years.

### Clinical Vignette

Progression of COPD: The following is a clinical vignette based on a patient recruited to the ARCADE study.

A 69-year-old male past smoker with a pack history of 40 pack years presented with mild breathlessness walking uphill (modified Medical Research Council (mMRC) 1).

Baseline assessments were as follows:

- Spirometry: FEV_1_ 2.15, forced vital capacity (FVC) 4.4, FEV1:FVC 0.49 (L). FEV_1_ percent of predicted 67%;
- Clinical measures: BMI 27.7 kg/m^2^, 6-min walk distance 450 m, high sensitivity c-reactive protein (HsCRP) 1.6 mg/L;
- Cardiovascular measures: Blood pressure 133/78 mmHg, aortic PWV 8.9 m/s.

After two years, the assessments were as follows:

- Spirometry: FEV_1_ 1.97, FVC 3.8, FEV1:FVC 0.52 (L). FEV_1_ percent of predicted 63%;
- Clinical measures: BMI 30.4 kg/m^2^, 6-min walk distance 405 m, HsCRP 1.5 mg/L;
- Cardiovascular measures: Blood pressure 137/82 mmHg, aortic PWV 9.4 m/s.

This case study describes a patient who was relatively clinically stable, with a small decline in lung function after two years. Despite a small change in blood pressure, which remained normotensive, there was an increase in aortic PWV of 0.5 m/s, indicating greater arterial stiffness and increased cardiovascular risk.

## 2. Materials and Methods

### 2.1. Design and Participants

The ARCADE is a prospective longitudinal observational study of patients with prior physician-diagnosed COPD and comparators undertaken in Cardiff, U.K. (NCT 01656421). The study was conducted in accordance with the Declaration of Helsinki and Good Clinical Practice Guidelines and was approved by the South East Wales Ethics Committee, which is part of the National Research Ethics Service (U.K.).

Patients had COPD, confirmed by postbronchodilator spirometry, and were clinically stable (at least four weeks from exacerbation). Patients with other inflammatory disease or on long-term oxygen therapy were excluded to avoid the known cardiovascular complications and possible effects on the arterial wall and amplification of cardiovascular risk. Comparators were current or past smokers with a spirometry of FEV_1_/FVC > 0.7. All participants were aged 35–80 years at baseline and continued to receive their regular prescribed medications throughout the study.

**Figure 1:**
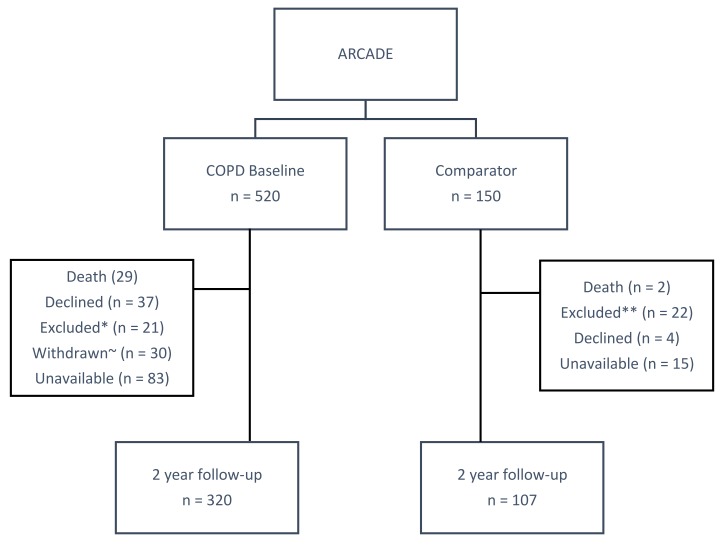
Flow diagram of recruitment and follow-up of participants. * Chronic obstructive pulmonary disease (COPD) exclusions: Cancer (*n* = 16), long-term oxygen therapy (*n* = 4), dementia (*n* = 1); ~ COPD withdrawn: Unwell and unable to attend the second visit; ** comparator exclusions: Cancer (*n* = 15), respiratory disease (*n* = 5), aortic aneurism (*n* = 1), myocardial infarction (*n* = 1).

Recruitment sources included general practice databases, respiratory outpatient clinics, pulmonary rehabilitation and smoking cessation referrals, and previous participants in respiratory research at Cardiff University. Participants were assessed at baseline and after two years. The recruitment and follow-up of participants is illustrated in Figure 1.

### 2.2. Data Collection

The following outcome measures were completed at baseline and repeated after two years, where possible. Mortality data including cause of death were collected from the National Health Service (NHS) Information Service Centre. The full methods, including the determination of acceptable reproducibility of haemodynamic measures, have been published [13]. The following assessments were included in the current analysis.

### 2.3. Anthropometry and Pulmonary Function

Height and weight were determined barefoot and used to calculate body mass index (BMI). A postbronchodilator spirometry was performed after inhalation of salbutamol (200 µg) (Vitalograph, Buckingham, U.K.). Forced expiratory volume (FEV_1_), forced vital capacity (FVC), and their ratio (FEV_1_/FVC) were noted [14].

Patients completed a questionnaire that included medical and smoking history, modified medical research council (mMRC) breathlessness and number of exacerbations per year.

### 2.4. Physical Performance

Six-minute walking distance (6-MWD) with pre- and post-test resting heart rate (HR) and oxygen saturation (SaO_2_%) were recorded by pulse oximetry according to guidelines [15].

### 2.5. Cardiovascular Measurements

After 10 min of rest, seated and supine peripheral systolic and diastolic blood pressure (BP) were determined (OMRON Corporation, Kyoto, Japan). Arterial waveforms were recorded with a high-fidelity micromanometer (SPC-301, Millar Instruments, Houston, TX, USA). A corresponding central (aortic) waveform was generated using the SphygmoCor system (AtCor Medical, Sydney, Australia) [16]. Central systolic and diastolic pressure and mean arterial pressure were calculated from the central aortic waveform. The pulse wave velocity (PWV) was measured using the same device by sequentially recording electrocardiogram-gated carotid and femoral artery waveforms (aortic PWV).

Cardiac output, stroke volume, and cardiac index were non-invasively determined using a chest bioreactance technique (Non-Invasive Cardiac Output Monitoring (NICOM™) (Cheetah Medical, Wilmington, DE, U.S.)) [17].

### 2.6. Blood Biochemistry

Venous blood was obtained to determine systemic inflammation (fibrinogen and high-sensitivity c-reactive protein (Hs-CRP) in the Cardiff and Vale University Hospital biochemistry laboratory according to standard procedures. Fibrinogen was included because of its value in identifying high-risk COPD patients both in clinical practice and due to its association with phenotypes in COPD [18]. Hs-CRP was chosen given its prognostic value in cardiovascular disease [19].

### 2.7. Statistical Analysis

Clinical and demographic data were expressed as mean ± SD or median (interquartile range (IQR)) for nonparametric data. The baseline parametric analysis included an independent *t*-test, with the Mann–Whitney *U* test and chi-square test being used for nonparametric data. Where appropriate, positively skewed data were log_10_-transformed for analysis and expressed as geometric mean ± SD. Baseline aPWV was additionally adjusted for age, mean arterial pressure (MAP), and HR using analysis of covariance when comparing aPWV between COPD patients and comparators. Univariable linear regression models were fitted to identify factors associated with aPWV. Explanatory variables with a *p*-value ≤ 0.15 and not highly correlated with another variable of interest were used to build a stepwise multivariable model, and aPWV was log-transformed in order to fulfill the model assumption of linearity. Longitudinal data were analyzed using repeated measures ANOVA, with differences in baseline and two-year follow-up measurements expressed as the mean (95% confidence interval (CI)). The analysis was undertaken using the statistical software package SPSS 20.0 (Chicago, IL, U.S.).

## 3. Results

### 3.1. Demographics of Baseline Recruitment

At baseline, 520 patients with COPD and 150 comparators were assessed. Patients with COPD and comparators were similar in age, gender, and BMI (Table 1). However, COPD patients had a greater smoking history, and there were more current smokers than in the comparators (*p* < 0.05). COPD patients had poorer lung function according to spirometry and lower 6-MWDs and resting SaO_2_ than comparators (*p* < 0.05) (Table 1). The majority of patients had moderate to severe disease according to the Global Initiative for Chronic Obstructive Lung Disease (GOLD) categories: GOLD 1 (*n* = 70), GOLD 2 (*n* = 269), GOLD 3 (*n* = 146), GOLD 4 (*n* = 35) [20].

**Figure 2:**
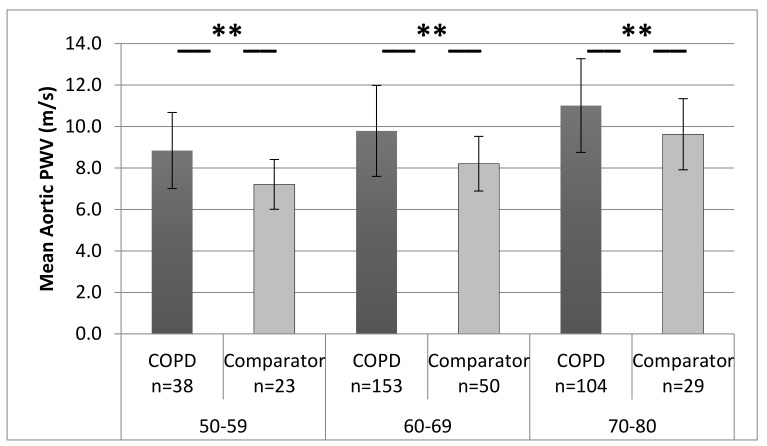
Mean (standard deviation) aPWV in patients with COPD and comparators across each decade. ** = *p* < 0.001 significant difference between groups; note: age <49 excluded due to small numbers.

Patients with COPD had a greater aPWV, peripheral systolic BP, central systolic and diastolic BP, MAP, and heart rate, and had a lower stroke volume (*p* < 0.01), than comparators. Inflammation (fibrinogen and HsCRP) was also greater in COPD patients. The greater aPWV in COPD patients remained after adjustments for age, MAP, and heart rate: COPD patients 9.8 (95% CI 9.7–10) versus comparator 8.7 (8.5–9.1) m/s (*p* < 0.01). The aPWV was greater in patients than for comparators in each age decade (<49 years excluded due to small numbers) (*p* < 0.05) (Figure 2).

### 3.2. Medical History

Patients with COPD reported more comorbidities: Hypertension and hypercholesterolemia were more frequent in COPD patients than in comparators (Table 2). A total of 84% (*n* = 437) of COPD patients reported one or more comorbidities, compared to 66% (*n* = 89) of comparators (*p* < 0.05). Patients were also prescribed more medications than comparators (*p* < 0.05) (Table 2). In the patient group, inhaled therapy comprised short-acting β_2_-agonists (*n* = 320), long-acting β-agonists (LABA) (*n* = 31), long-acting muscarinic antagonists (*n* = 305), inhaled corticosteroids (ICS) alone (*n* = 29), combination (ICS & LABA) (*n* = 275), anticholinergics (*n* = 12), and methylxanthines *n* = 15. None of the comparators received inhaled therapy.

#### Determinants of Aortic PWV

In COPD patients, gender, age, FEV_1_/FVC, BMI, central MAP, HR, 6-MWD, SaO_2_, smoking status, and the number of comorbidities were related to aPWV_log10_ (*p* < 0.15). After adjustment, among COPD patients, age, BMI, MAP, and the number of comorbidities were significantly associated with aPWV_log10_ (*p* < 0.05). Similarly, among comparators, after adjustment, age, MAP, and HR were significantly associated with aPWV_log10_ (Table 3). The back-transformed regression coefficient represents the proportional increase in aPWV for each unit change of the corresponding explanatory variable. For example, for COPD patients, for a 1-yr increase in age, aPWV increased by a factor of 1.013, i.e., by 1.3%.

### 3.3. Follow-Up Data

At the two-year follow-up, 320 patients and 107 comparators returned for reassessment (Figure 2). Complete CV datasets were available for 301 patients and 105 comparators. A comparison of participants that completed and did not complete the visit showed similar baseline characteristics (Supplementary Materials, Table S1).

Patients and comparators had a slight decline in FEV_1_ (L) and FVC (L), but no change in FEV_1_/FVC or FEV_1_% predicted. Among COPD patients, there was a significant decline in the mean (95% CI) 6-MWD of 27 m (15–39 m), and a 12 m (3–27 m) decline among comparators (*p* < 0.05) (Table 4). Both COPD patients and comparators had a similar increase in aPWV of almost 0.5 m/s over two years (*p* < 0.05), despite a mean decrease in peripheral systolic BP and no change in central MAP, cardiac output (CO), stroke volume (SV), or inflammatory markers. There was a variable pattern of change in other variables among patients with COPD: 64% had a decline in FEV_1_ (L), 56% had a decrease in 6-MWD, 58% had an increase in aPWV, and 42% had an increase in systolic and diastolic BP.

### 3.4. Mortality

After two years, there were 29 deaths (6%) in COPD patients and 2 deaths (1%) in the comparator group. The causes of mortality in COPD patients were respiratory (*n* = 13), cardiovascular (*n* = 2), cancer (*n* = 6), and other (*n* = 8). As expected, COPD patients with more severe disease (FEV_1_ < 50%) had greater mortality (*n* = 17 (10%)) than patients with milder disease (FEV_1_ > 50%, *n* = 12, (4%)) (*p* < 0.05).

## 4. Discussion

The ARCADE study is the first large prospective longitudinal study of a well-characterized cohort of patients with COPD and comparators free from lung disease, with a focus on aPWV as a measure of CV risk. The novelty of the ARCADE is the evaluation of the predictive role of aPWV, an important indicator of cardiovascular risk [9], which has not been included in other longitudinal studies of COPD [21,22].

The baseline results of our study demonstrated aortic PWV was greater in the patients than the comparators, confirming previous smaller cross-sectional studies [10,23]. The difference was 1.6 m/s, which represents a substantial increase in risk in the context of the meta-analysis that showed that a 1 m/s increase represents a 15% increase in CV risk [9]. Systolic blood pressure and central MAP were greater in the COPD patients than in the comparator subjects, which may have been a cause or consequence of central vascular stiffness. Increased arterial stiffness and wave reflections, along with changes in central haemodynamics, increase left ventricle afterload, enhance the left ventricular remodeling process, and also decrease the coronary blood flow, resulting in subendocardial ischemia and myocardial injury [12,24,25]. This may result in subclinical CV disease, including left ventricular dysfunction, and may be interpreted as premature vascular aging [10,12]. The presence of an increased heart rate and lower stroke volume in patients may have been a consequence of cardiac dysfunction, but also may have been linked to ventilatory dynamics [26].

As expected, lung function was impaired in COPD patients, and patients were more breathless and had lower exercise capacity (6-MWD) than comparators. Although biomarkers of systemic inflammation, HsCRP, and fibrinogen were greater in the patient group, it is of note that mean fibrinogen was within normal values (3.5 g/L) [18], and inflammation was relatively high in comparators: This may be explained by the age and presence of subclinical disease in some of the comparator subjects. The mean value of HsCRP was substantially elevated in COPD patients, indicating high CV risk according to published studies, but was normal in comparators [27].

At baseline, in patients and comparators, aPWV was associated with age, MAP, and HR, with BMI and the number of comorbidities additionally associated with COPD. The relationship between arterial stiffness and age, blood pressure, and HR has been well established and is potentially a consequence of increased pulsatile stress promoting vascular remodeling and arterial stiffness [28]. There have been conflicting results regarding the relationship between aPWV and lung function and inflammation in COPD. One study showed FEV_1_ was a predictor of aPWV in patients with COPD without CV disease, but found that CRP was unrelated [29]. Meanwhile, Sabit et al. have reported that aPWV was related to FEV_1_ and inflammation (interleukin-6) in COPD [10]. It has been suggested that vascular inflammation has a role in the development of increased aortic stiffness [30], while BMI is associated with metabolic abnormalities/comorbidities, which may explain the contribution of both to the model [31]. After two years, the mean change in aPWV in patients and comparators was similar, with an increase of almost 0.5 m/s. This is similar to a previous study that showed an annual increase of 0.24 m/s in older (>50 years) treated hypertensives [32]. Although the mean increase in aPWV over two years was modest, representing a less than 10% increase in CV risk [9], there was a variable pattern of change among participants. Nearly 60% of patients and comparators had an increase in aPWV, while 39% had a decrease. In neither group was the change correlated to baseline parameters, and therefore we were unable to identify particular characteristics of individuals at risk of increased aPWV. Previous research has linked changes in PWV to changes in blood pressure, but the lack of association in the present study may have been a consequence of the optimization of blood pressure when the results were communicated to primary care physicians.

There was a variable pattern of change over two years in other measures of COPD disease severity, including in lung function and 6-MWD. Although the majority of patients with COPD and comparators had a decline in lung function and a reduction in 6-MWD, a smaller percentage had an increase, and some individuals did not change over two years. This heterogeneity aligns with previous longitudinal studies that found that some patients had stable lung function [33] and 6-MWDs [34] for a number of years. Both patients and comparators showed a decline in lung function over two years. A decline in absolute FEV_1_ (L) would be expected with aging, but the reduction in FEV_1_% predicted likely reflected past exposure to risk factors such as smoking in both groups. The reduction in 6-MWD in COPD patients only could be attributed to further reductions in the already impaired lung function in COPD patients and the resulting reduction of activity and exercise capacity, while the comparators, although losing some lung function, remained within normal limits (which may explain their ability to maintain their 6-MWD).

### 4.1. Mortality

At the two-year follow-up, the mortality rate in COPD patients was 6%, with the majority due to pulmonary causes. Mortality was greater in COPD patients than in the comparators (1%) and was not dissimilar to the 10% mortality reported in the Evaluation of COPD Longitudinally to Identify Predictive Surrogate End-points ECLIPSE study, after three years [35]. However, the number of deaths of cardiovascular origin was low, which could be explained by the fact that many patients were treated with antihypertensives and statins to minimize CV risk factors. Aortic PWV is a predictor of CV events and mortality in the general population, but it has shown only a modest correlation with lung function in previous studies and was unrelated to FEV_1_ in this study. This may explain why it would be unlikely to predict lung-related mortality. A longer-term follow-up may yet establish its utility as a predictor for CV risk in COPD patients.

### 4.2. Strengths and Limitations

ARCADE is the first large longitudinal study of COPD patients, and includes cardiovascular risk factors alongside traditional markers of disease such as lung function, reported exacerbations, breathlessness, and exercise capacity. The results were consistent with previous data [33] and are likely to be representative of community-dwelling patients with COPD across the spectrum, from mild to severe disease (all with spirometric evidence of airway obstruction). The presented data were limited by differences in smoking status and overt CV disease between the groups. This demonstrates the difficulty in identifying healthy smoker comparators. In order to recruit a representative group of patients with COPD, we did not exclude overt CV disease from the COPD group, but to minimize the effect of confounders, we excluded other inflammatory states, such as rheumatoid arthritis and cancer, in patients and comparators.

There was a greater loss to follow-up at two years in patients with COPD (43%) than in comparators (30%), which may have been a consequence of poorer health (greater exclusions) and an inability or unwillingness to return for the second assessment. However, overall participants who completed the study were similar in baseline characteristics to those who failed to complete the two-year assessment. Additional missing data analysis may have provided additional information but was beyond the scope of this paper.

The dropout rate was approximately double that of the ECLIPSE study, which reported 18% dropout over two years. However, the ECLIPSE study included follow-ups at three and six months, which may have retained participants [21], and a more recent longitudinal study of patients with COPD (GOLD B) reported a two-year dropout rate of 63% [36].

In epidemiological studies, dropout rates have ranged between 30% and 70%, with high dropout associated with smoking and poor socioeconomic status [37]. This may explain the high attrition in patients compared to comparators. The mortality data were based on local NHS records, so there was potential for some missing data: However, this was minimized, as we attempted to contact all participants for the follow-up and received some information from their relatives.

## 5. Conclusions

ARCADE is the first large longitudinal study of CV risk in COPD patients, and it confirmed greater arterial stiffness in COPD than comparators. Increased aPWV was observed in COPD patients across all age groups, and although the rate of increase was similar to the controls, this suggests early or more rapid onset of arterial stiffening in COPD patients. A continued follow-up of ARCADE participants will investigate predictors of mortality.

## 6. Clinical Trials

This study is registered at ClinicalTrials.gov with the identification number NCT01656421.

## Figures and Tables

**Table 1:** Baseline characteristics of patients with COPD and comparators.

	COPD, *n* = 520	Comparators, *n* = 150	*p*-Value
Age (years)	66.1 ± 7.6	65 ± 7.4	0.109
Gender, male/female, *n*	270:250	76:74	0.786
BMI (Kg/m^2^)	28 ± 5.5	28.1 ± 4.1	0.942
FEV_1_ (L)	1.42 ± 0.60	2.74 ± 0.68	<0.001
FVC (L)	2.67 ± 0.90	3.53 ± 0.90	<0.001
FEV_1_/FVC (L)	0.53 ± 0.11	0.78 ± 0.05	<0.001
FEV_1_ (% of predicted)	58 ± 19	105 ± 14	<0.001
FVC (% of predicted)	87 ± 21	109 ± 15	<0.001
Smoking (pack years)	40 ± 26	21 ± 18	<0.001
Current smokers, *n* (%)	183 (35%)	26 (17%)	<0.001
mMRC (median (IQR))	2 [1–3]	-	-
No. Exac/Yr (median [IQR])	2 [1–3]	-	-
Resting O_2_ (%)	97 ± 2	98 ± 1	<0.001
6-MWD (m)	335 ± 125	502 ± 86	<0.001
aPWV (m/s)	10 ± 2.4	8.4 ± 1.8	<0.001
Peripheral SBP (mmHg)	146 ± 18	140 ± 18	<0.001
Peripheral DBP (mmHg)	82 ± 10	81 ± 9	0.{Patel, 2006 #110}3
Peripheral PP (mmHg)	64 ± 16	60 ± 15	0.001
Central SBP (mmHg)	127 ± 17	123 ± 16	0.020
Central DBP (mmHg)	77 ± 10	74 ± 9	0.005
Central PP (mmHg)	50 ± 13	49 ± 12	0.335
Central MAP (mmHg)	98 ± 11	95 ± 10	0.004
Heart rate (bpm)	74 ± 11	67 ± 11	<0.001
Cardiac output (L/min)	5.8 ± 1.2	6.0 ± 1.1	0.165
Cardiac index (L/min/m^2^)	3.2 ± 0.5	3.2 ± 0.5	0.864
Stroke volume (mLs)	86 ± 20	96 ± 20	<0.001
Fibrinogen (g/L) #	3.5 {3.4, 3.6}	3.1 ± {2.9, 3.2}	<0.001
HsCRP (mg/L) #	3.4 {3.1, 3.8}	1.7 ± {1.4, 2.1}	<0.001

Data are mean ± SD unless stated; # geometric mean (95% confidence interval); *p* < 0.05, significant difference between groups; aPWV = aortic pulse wave velocity; 6-MWD = six-minute walk distance; BMI = body mass index; FEV_1_ = forced expiratory volume in 1 s; FVC = forced vital capacity; HsCRP = high sensitivity c-reactive protein; mMRC = modified medical research council breathlessness; IQR = interquartile range; SBP = systolic blood pressure; DBP = diastolic blood pressure; PP = pulse pressure; MAP = mean arterial pressure.

**Table 2:** Comorbidities and medications in patients with COPD and comparators.

	COPD, *n* = 520	Comparators, *n* = 150	*p*-Value
No. of comorbidities	3 [2–4]	2 [1–3]	<0.001
Hypertension	245 (47%)	34 (23%)	<0.001
Angina	61 (12%)	0 (0%)	<0.001
Myocardial Infarction	46 (9%)	0 (0%)	0.002
Atrial Fibrillation	42 (8%)	5 (3%)	0.018
Heart failure	19 (4%)	0 (0%)	0.025
Other heart diseases $	40 (8%)	4 (3%)	0.045
Transient ischaemic attack	36 (7%)	2 (1%)	0.009
Hypercholesterolemia	237 (46%)	41 (27%)	<0.001
Diabetes mellitus	67 (13%)	0 (0%)	<0.001
Osteoporosis	87 (17%)	9 (6%)	0.001
Peripheral vascular disease	19 (4%)	3 (2%)	0.317
Osteoarthritis	177 (34%)	40 (27%)	0.089
No. of medications	5 [3–8]	2 [0–3]	<0.001
Angiotensin-converting enzyme inhibitors	115 (22%)	9 (6%)	<0.001
Angiotensin receptor blockers	44 (8%)	2 (1%)	0.002
Calcium channel blockers	114 (22%)	8 (5%)	<0.001
Beta blockers	42 (8%)	5 (3%)	0.047
Diuretics	109 (21%)	11 (7%)	<0.001
Anticoagulants	27 (5%)	2 (1%)	0.041
Statins	192 (37%)	27 (18%)	<0.001
Bisphosphonates	72 (14%)	5 (3%)	<0.001
Inhaler therapy	419 (81%)	0 (0%)	<0.001

Note: *n* (%) or median (IQR); *-* not assessed; *p* < 0.05 significant difference between groups; $, other heart disease = arrhythmia, enlarged heart, heart murmur, valve disease, or vessel disease; inhalers = bronchodilators, inhaled corticosteroids, or antimuscarinics, alone or in combination.

**Table 3:** Factors associated with aPWV_log10_ among COPD patients and comparators.

	Regression Coefficient, Beta (S.E.)	Back-Transformed Regression Coefficient *	*p*-Value
COPD (*n* = 520)			
Age (years)	0.0055 (0.001)	1.013	<0.001
Central MAP (mmHg)	0.0023 (0.000)	1.005	<0.001
HR (bpm)	0.0020 (0.000)	1.005	<0.001
BMI (Kg/m^2^)	0.0025 (0.001)	1.006	<0.001
No. of comorbidities	0.0085 (0.003)	1.019	0.001
Comparator (*n* = 150)			
Age (years)	0.0053 (0.001)	1.012	<0.001
Central MAP (mmHg)	0.0025 (0.001)	1.006	<0.001
HR (bpm)	0.0023 (0.001)	1.005	0.001

BMI = body mass index; HR = heart rate; MAP = mean arterial pressure; S.E. = standard error; * back-transformed regression coefficient was calculated by 10^ beta.

**Table 4:** Baseline and two-year follow-up lung function, haemodynamic data, and exercise capacity in COPD patients and comparators.

	COPD (*n* = 301)			Comparators (*n* = 105)		*p*-Value
	Baseline	Two Years	*p* =	Baseline	Two Years	
FEV_1_ (L)	1.41 ± 0.59	1.34 ± 0.60	<0.001	2.73 ± 0.69	2.63 ± 0.69	<0.001
FVC (L)	2.68 ± 0.88	2.49 ± 0.91	<0.001	3.48 ± 0.91	3.37 ± 0.94	0.001
FEV_1_ FVC (L)	0.52 ± 0.12	0.54 ± 0.13	0.001	0.79 ± 0.05	0.78 ± 0.05	0.387
FEV_1_ %	58 ± 19	57 ± 21	0.174	106 ± 14	104 ± 19	0.124
SBP (mmHg)	147 ± 19	143 ± 19	<0.001	140 ± 18	137 ± 16	0.032
DBP (mmHg)	82 ± 10	80 ± 10	<0.001	80 ± 9	80 ± 8	0.294
Central MAP (mmHg)	98 ± 12	98 ± 11	0.208	95 ± 11	94 ± 11	0.775
Aortic PWV (m/s)	10 ± 2.3	10.5 ± 2.6	<0.001	8.3 ± 1.7	8.8 ± 1.8	0.000
Cardiac output (L/min)/#	5.8 ± 1.2	5.9 ± 1.3	0.084	5.9 ± 1.1	6.1 ± 1.4	0.109
Cardiac index (L/min/m^2^) #	3.2 ± 0.5	3.3 ± 0.6	0.005	3.1 ± 0.5	7.6 ± 39.5	0.250
Stroke volume (mLs) #	87 ± 20	87± 22	0.882	95 ± 21	99 ± 24	0.063
6-MWD (m) ~	359 ± 110	332 ± 149	<0.001	508 ± 83	496 ± 80	0.107

Data are mean ± SD; # = COPD (*n* = 284); ~ = COPD (*n* = 264); comparator (*n* = 100); *p* < 0.05 significant difference within groups; FEV_1_ = forced expiratory volume in 1 s; FVC = forced vital capacity; PWV = pulse wave velocity; SBP = systolic blood pressure; DBP = diastolic blood pressure; PP = pulse pressure; MAP = mean arterial pressure; 6-MWD = six-minute walk distance.

## Supplementary Materials

The following are available online at https://www.mdpi.com/1010-660X/55/4/89/s1, Table S1. Baseline Characteristics of Patients with COPD and Comparators who completed or did not complete the 2 year assessment.
