# Supplementary material for: Aortic Pulse Wave Velocity as a Measure of Cardiovascular Risk in Chronic Obstructive Pulmonary Disease: Two-Year Follow-Up Data from the ARCADE Study
Source: Medicina (Kaunas). 2019 Apr 2;55(4):89. doi: 10.3390/medicina55040089 (PMC6524022; doi:10.3390/medicina55040089)
Supplement: Supplementary file 1 [file medicina-55-00089-s001.pdf]

Supplementary data

Table S1. Baseline Characteristics of Patients with COPD and Comparators who completed or did not complete the 2 year assessment.

	**COPD complete n=301**	**COPD non-complete n=219**	**Comparator complete n=105**	**Comparator non-complete n=45**
Age (years)	66.4 ± 7.1	65.7 ± 8.2	66 ± 7	64 ± 8
Male n (%)	156 (51.8%)	114 (52.1%)	54 (51.4%)	22 (48.9%)
BMI (Kg/m^2)	27.8 ± 5.2	28.4 ± 6.0	28.0 ± 4.0	28.2 ± 4.5
FEV_1 (L)	1.41 ± 0.59	1.44 ± 0.61	2.75 ± 0.71	2.72 ± 0.59
FVC (L)	2.69 ± 0.89	2.65 ± 0.92	3.52 ± 0.96	3.54 ± 0.75
FEV_1/FVC (L)	0.52 ± 0.12	0.54 ± 0.10	0.79 ± 0.05	0.77 ± 0.05
FEV_1(% of predicted)	57 ± 19	59 ± 19	106 ± 14	104 ± 12
FVC (% of predicted)	87 ± 21	86 ± 21	109 ± 16	110 ± 14
Smoking (pack years)	40 ± 27	41 ± 23	21 ± 18	23 ± 20
Current smokers, n (%)	88 (29%)	95 (43%)	18 (17%)	8(17%)
mMRC (median [IQR])	2 [1-3]	2 [1-3]	-	-
No.Exac Yr (median [IQR])	2 [1-3]	2 [1-3]	-	-
Resting O_2 (%)	97 ± 2	97 ± 2	98 ± 1	98 ± 1
6MWD (m)	350 ± 116	313 ± 134	505 ±83	496 ± 92
aPWV (m/s)	10.0 ± 2.3	9.9 ± 2.5	8.4 ± 1.7	8.5 ± 2.1
Peripheral SBP (mmHg)	147 ± 19	145 ± 18	140 ±18	141 ± 19
Peripheral DBP (mmHg)	82 ± 10	82 ± 11	80 ± 9	82 ± 7
Central MAP (mmHg)	98 ± 11	97 ± 11	95 ± 11	95 ± 9
Heart Rate (bpm)	74 ± 11	75 ± 12	67 ± 10	69 ± 11
Fibrinogen (g/L) #	3.5 ± 1.3	3.5 ± 1.3	3.1 ± 1.3	3.0 ± 1.3
HsCRP (mg/L) #	3.1 ± 3.0	3.9± 2.9	1.8 ± 3.0	1.5 ± 3.4

Data are Mean ±SD unless stated, # geometric mean $p<0.05$ significant difference between groups
aPWV= aortic pulse wave velocity, 6MWD= six minute walk distance, BMI= body mass index, FEV_1, forced expiratory volume in 1 second, FVC= forced vital capacity, mMRC= modified medical research council breathlessness, SBP= systolic blood pressure, DBP= diastolic blood pressure, pressure, MAP= mean arterial pressure.
